# Design and Rationale for a Parent-Led Intervention to Increase Fruit and Vegetable Intake in Young Childhood Cancer Survivors (Reboot): Protocol for a Pilot Study

**DOI:** 10.2196/resprot.9252

**Published:** 2018-05-16

**Authors:** Lauren Touyz, Jennifer Cohen, Claire Wakefield, Allison Grech, Sarah Garnett, Paayal Gohil, Richard Cohn

**Affiliations:** ^1^ Behavioural Sciences Unit, Kids Cancer Centre School of Women's and Children's Health University of New South Wales Sydney Sydney Australia; ^2^ Institute of Endocrinology and Diabetes The Children's Hospital at Westmead Sydney Australia; ^3^ Behavioural Sciences Unit, Kids Cancer Centre School of Women's and Chidlren's Health University of New South Wales Sydney Sydney Australia

**Keywords:** childhood cancer survivor, child, diet, feeding patterns, fruit, vegetables

## Abstract

**Background:**

Poor dietary habits are common among childhood cancer survivors, despite increasing their risk of cardio metabolic complications after cancer treatment. Here, we describe the design and rationale for a pilot telephone-based, parent-led intervention aimed at increasing fruit and vegetable intake in young cancer survivors (Reboot).

**Objective:**

This pilot study aims to assess the feasibility and acceptability of delivering evidence-based telephone support to parents of childhood cancer survivors. A secondary aim includes assessing the effect of Reboot on improving childhood cancer survivors’ dietary quality by increasing child fruit and vegetable intake and variety and its contribution to overall nutrient intake.

**Methods:**

We aim to recruit parents of 15 young cancer survivors aged 2 to 12 years who have completed cancer treatment less than five years ago. The intervention comprises of 4 weekly 45-minute telephone sessions led by a health professional and one booster session 6 weeks later. Sessions address the effects of cancer treatment on children’s diets, recommended fruit and vegetable intake for children, and evidence-based strategies to promote the consumption of fruit and vegetables as well as to manage fussy eating.

**Results:**

Reboot is based on an existing, evidence-based parent nutrition intervention and modified for childhood cancer survivors following extensive collaboration with experts in the field. Primary outcomes of feasibility and acceptability will be measured by the number of participants who complete all five sessions, average session length (minutes), length between sessions (days) and parent Likert ratings of the usefulness and impact of the intervention collected after the booster session. Of the 15 participants we aim to recruit, 3 have completed the intervention, 1 declined to participate, 11 are actively completing the intervention and 2 participants are providing written consent. The remaining 3 participants will be recruited via telephone follow-up calls. The intervention is due to be completed by July 2018.

**Conclusions:**

Reboot aims to support healthy dietary behaviors in childhood cancer survivors who are at increased risk of developing serious cardiometabolic complications after their cancer treatment. Results will inform the development and implementation of future evidence-based dietary interventions delivered to childhood cancer survivors, particularly those living in rural and remote areas.

**Registered Report Identifier:**

RR1-10.2196/9252

## Introduction

The cardiotoxic effects of chemotherapy and radiation have been shown to contribute to an increased risk of cardiovascular disease in survivors of childhood cancer [1]. Consequently, childhood cancer survivors (CCS) are more than six times more likely to experience serious cardiac conditions compared with their siblings, with rates continuing to increase as late as thirty years after cancer treatment [2].

One of the key predisposing factors for cardiovascular disease is metabolic syndrome, a cluster of conditions which includes central obesity, dyslipidemia, glucose intolerance and hypertension [3]. There is a strong association between a diet low in fruit and vegetables and high in saturated fat and sugar, and the onset of metabolic syndrome in CCS [4]. Consequently, CCS with metabolic syndrome are twice less likely to meet national dietary guidelines compared with those who do not have the syndrome [4].

Despite their increased risk of developing serious chronic health conditions, young CCS often report poor health-protecting behaviors [5-8] (eg, reduced intake of fruit and vegetables, excessive energy, and inadequate calcium and folate intake) [9] and a higher intake of non-core (“junk”) foods compared with prediagnosis [10]. These behaviors often develop during treatment when children’s home food environment and eating are disrupted by frequent hospital admissions and treatment related side-effects including nausea, increased appetite, and vomiting [11]. Unhealthy eating habits established during cancer treatment also appear to be exacerbated by parents of CCS who report using unhealthy food as a means to reduce children’s pain and emotional distress after cancer treatment [10,11].

Although dietary behaviors are among the most easily modifiable factors for reducing the risk of cardio metabolic complications, CCS and their families often live long distances from their tertiary hospital [12] making it difficult for health professionals to provide ongoing nutritional support after treatment completion. Telephone interventions may therefore represent a feasible and acceptable approach for promoting healthy eating habits among CCS.

Telephone-based parent-led interventions have successfully increased fruit and vegetable intake in children not previously treated for cancer [13-17]. However, the feasibility and acceptability of telephone-based, parent-led fruit and vegetable interventions in CCS is yet to be evaluated. Therefore, we aim to assess the feasibility and acceptability of delivering evidence-based telephone nutritional support to parents of CCS.

## Methods

### Recruitment

We aim to recruit the parents of 15 CCS. This number is sufficient to provide exploratory findings to inform the design of a randomized controlled trial (RCT) in this population [18]. Based on our previous research, we assume a response rate of 50% and attrition rate of 20% [18-20], and therefore it was anticipated that approximately 50 parents will need to be contacted to recruit 15 families.

Parents will be eligible to participate if they have a child aged between 2-12 years who completed cancer treatment at the Sydney Children’s Hospital (SCH), Randwick, NSW, with curative intent, achieved remission less than 5 years ago and meet the following criteria which are (1) to provide informed consent, (2) be able to read English, and (3) have regular telephone access. Parents will be ineligible if they have (1) insufficient English language skills to complete telephone sessions and or baseline and follow-up assessments, (2) severe depression or suicidal ideation, as determined by the clinical experience of the treating oncologist, or if their child who had cancer, (3) is currently on active treatment or receiving supplementary feeding, (4) has relapsed, (5) is in palliative care or is deceased, or (6) completed cancer treatment more than five years ago.

Eligible participants will be recruited via mail using an information and consent form explaining the purpose of the project and details of participation. Participants can choose to participate by returning the consent form or by contacting the study coordinator. Nonrespondents will be contacted by study personnel via telephone or text message. This protocol was approved by the Network Human Research Ethics Committee (HREC/15/SCHN/395).

### Intervention

Reboot models a previous intervention aimed at increasing fruit and vegetable intake in over 400 Australian children (who did not receive cancer treatment) [16]. Following a parent-led interventional model, the parent (not the child) receives the intervention and is responsible for regulating the home food environment and acting as an important role model, which maintains children’s eating behaviors [21].

**Figure 1:**
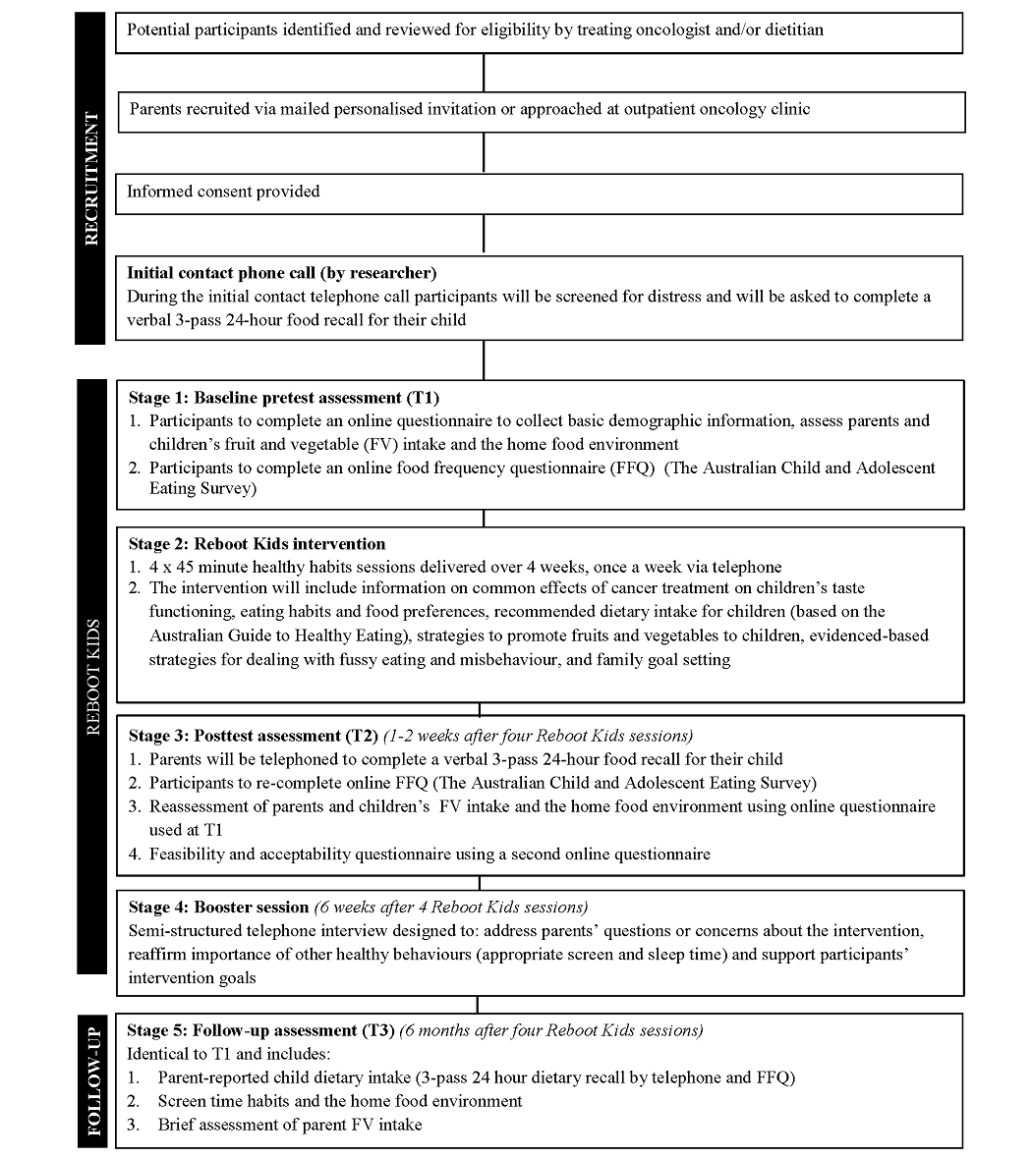
Reboot Kids study flowchart.

**Figure 2:**
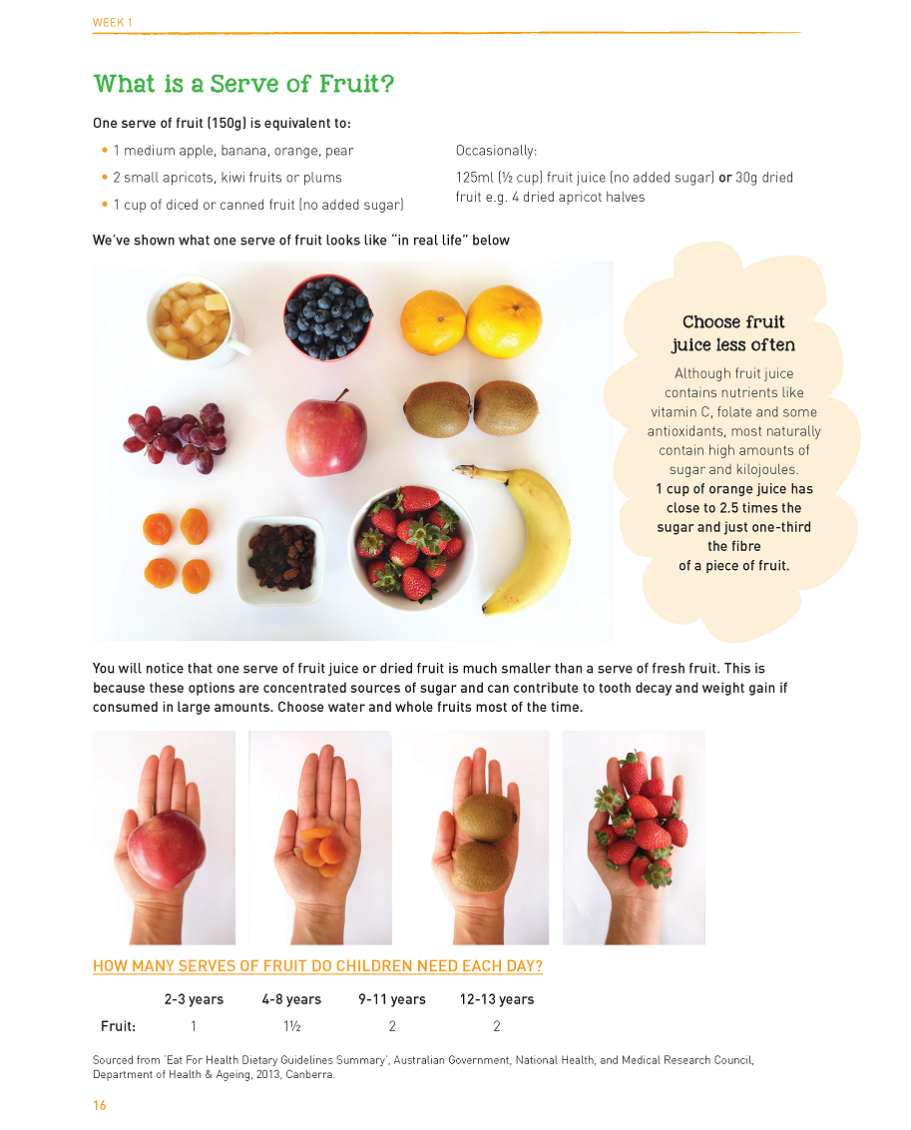
Excerpts from the Reboot Kids parent workbook.

In line with the original intervention, Reboot will be delivered to only one parent by a trained health professional (either a registered psychologist or dietitian) via 4 weekly 45-minute telephone sessions, with the addition of 1 booster session 6 weeks after the fourth intervention session (Figure 1). Intervention sessions will be guided by a parent guidebook (Figure 2) focusing on key factors associated with increased intake of fruit and vegetables in children, including the accessibility of fruit and vegetables in the home, parental providing and modelling of fruit and vegetable intake, and positive family-based mealtime practices (eg, eating together) [22]. Further details are shown in Table 1.

**Table 1:** Reboot intervention session objectives and cancer-relevant content (CCS: childhood cancer survivors).

Weekly session	Core objectives and skills	Cancer relevant content
Introduction and overview	Understanding the rationale for intervening or eliciting motivation. To promote mastery by normalizing parents’ concerns and fears. Understanding the Australian Guide to Healthy Eating and portion sizes. Information about the type and quantity of fruits and vegetables that children should be eating. Identify ‘non-core’ foods and strategies to reduce non-score food intake. Understanding the importance of parent providing fruit and vegetables throughout the day. Introduction of the parent vegetable providing diary to encourage parent self-monitoring of the number of occasions parents provide fruit and vegetables to children throughout the day. Setting specific and achievable program goals.	Importance of supporting healthy eating habits and regular physical activity after cancer treatment. Common experiences of children and parents during and after cancer treatment (eg, food aversions, poor fruit and vegetable intake, altered taste perceptions, physical inactivity, and parent overprotectiveness). End of cancer treatment is one of the most difficult times for families. Addressing challenges (eg, effects of treatment on re-establishing a normal routine, including fatigue, fussy eating, inconsistent discipline, misbehavior, and overprotectiveness).
The home food environment	Identify barriers to introducing change (establishing rules and coping with change). Tips for structuring family mealtimes. Providing praise and positive reinforcement. Brainstorm effective non-food rewards. Review strategies for creating a healthy home environment. Introduce meal planning. Plan for the week ahead.	Addressing challenges for families establishing change after cancer (eg, parent guilt and or overprotectiveness, misbehavior resulting from absence of discipline, and routine during treatment).
Encouraging children to eat vegetables	Practical strategies for promoting fruit and vegetables to children (eg, planning and providing choices). Tips on making food exciting and interesting. Preparing for misbehavior. Discussion of unhelpful strategies (what to try and what to avoid). Understanding the importance of role modelling healthy eating habits to children.	N/A
Consolidation	Review topics from the previous four weeks. Strategies for shopping with children. Review program goals. Preparing for future challenges.	Address any parent fears and or concerns moving forward.
Booster	Review program goals and progress (eg, CCS fruit and vegetable intake, parent-providing, and role modelling of fruit and vegetable intake to CCS). Introduce screen time guidelines. Introduce sleep guidelines. Harnessing support from family and friends.	Re-addressing challenges for families establishing behavior change after cancer, including other caregivers

### Treatment Late Effects

A study by our team identified a significant proportion (>90%) of Australian and New Zealand parents reporting unmet needs for information about the late effects of cancer treatment [23]. An introductory information module (“*Why is healthy eating important for children after cancer treatment?* ”) was developed to increase parents’ knowledge about the most common serious long-term health problems identified among childhood cancer survivors (eg, weight gain, heart disease, diabetes, high blood pressure, and high cholesterol) and evidence-based behaviors that may help to prevent or reduce occurrence of treatment late effects (eg, increasing children’s intake of fruits and vegetables).

### Changes in Parenting Behavior and Children’s Eating Habits

The introductory module also includes an overview about why healthy eating is difficult for children and parents after cancer treatment. A summary of the six most common challenges is provided and it includes topics such as loss of appetite, taste changes, food aversions, steroids, limited exposure to new foods, and increased parental leniency for unhealthy foods and emotional feeding. A synopsis about common changes in children’s eating habits after cancer treatment is also provided to normalize parents’ experiences (eg, increased intake of junk foods and decreased intake of vegetables).

Following our modifications, the parent guidebook was assessed for readability using the Flesch-Kincaid Grade Level Test [24], yielding a grade level test score of 7.6 or a seventh to eighth grade school level. In concordance with the Australian National Framework for Consumer Involvement in Cancer Control [25], parents of CCS (N=4) were involved in reviewing the guidebook and providing feedback prior to publication.

### Primary and Secondary Outcome Measures

#### Demographic Information

Information on parent sex, age, education and employment status will be collected at baseline (T1, see Figure 1) together with the sex and age of the CCS. A validated emotion thermometers tool will also be used to assess participant distress and emotional state at the start of each intervention session [26]. This tool individually screens for emotional upset, anxiety, depression, and anger by asking the participant to rate each emotion on a scale of 0-10 (where 0 is none and 10 is extreme), and the level of assistance required to manage these emotions [26]. A participant will be deemed “distressed” if they score above seven on any of the thermometers [20], requiring facilitators to enquire about the participants available psychological supports (eg, current psychologist, general practitioner [GP], or social worker), and, if needed, will provide information for appropriate services including a 24-hour crisis hotline. The chief investigator will be contacted, and a nominated health professional will be contacted with the participant’s consent if appropriate.

#### Feasibility and Acceptability

Feasibility will be assessed using several descriptive indices. These include the number of participants who complete all 5 intervention sessions (participation rate), average session length (minutes), and days between sessions. To evaluate the ease, usefulness, and impact of the intervention (see Textbox 1), participants will be invited to complete and online survey after the fourth intervention session (T2, Figure 1) to rate their agreement on several acceptability items using a 5-point Likert scale from strongly disagree to strongly agree (eg, “*The Reboot program improved my knowledge about strategies for promoting fruits and vegetables to my child* ”). Items from the task and goal subscales of the Validated Working Alliance Inventory were also included to evaluate participants perceptions of the facilitator (eg, competence) [27].

#### Childhood Cancer Survivors Fruit and Vegetable Intake

To measure CCS fruit and vegetable intake, participants will be invited to complete 2 validated dietary intake assessment methods at baseline (T1), after the fourth intervention session (T2) and 6 months after the fourth intervention session (T3) (see Figure 1). These measures will include the following aspects listed below.

### 24-hour Dietary Recall

A 24-hour dietary recall will be conducted by a registered dietitian via telephone [28] to obtain a list of everything the CCS ate and drank during the previous day. This information will be manually entered into FoodWorks 8, an Australian nutrient analysis software (Xyris, Brisbane, QLD, Australia), by a registered dietitian to produce a calculation of total daily fruit and total daily vegetable servings for each child [29]. Intake (total fruit and total vegetable servings consumed) will also be used to categorize CCS as meeting (or not meeting) the recommended daily intake for fruit and vegetables, separately, for their age.

### Food Frequency Questionnaire

The fruit and vegetable subscales scores from the Australian Child and Adolescent Eating Survey, a brief (15 minutes) online FFQ, will be used to provide quantitative data on the number of daily fruit and vegetable serves for age and variety of fruit and vegetables consumed [30-32] where a higher fruit (out of 12) and vegetable variety score (out of 21) indicates greater intake of a variety of fruit and vegetables [33].

### Statistical Analyses

Data will be analyzed using the Statistical Package for the Social Sciences, version 18.0 (SPSS Inc, Chicago, IL, USA). The primary outcome measure (number of participants who complete all 5 intervention sessions) will be analyzed using a repeated measures design. An 80% study compliance and 20% attrition rate [18] will be used as the benchmark for program feasibility. Cochran *Q* will be used to identify participants as meeting the specific study completion criterion. The secondary outcome measure is the difference in the mean number of fruit and vegetable serves consumed by CCS (on the previous day) from baseline to the end of the intervention (after the booster session). Paired *t*-tests will be conducted to determine whether there is a significant difference between the mean intakes of fruit and vegetable serves at baseline compared to the end of the intervention.

**Postintervention Acceptability Items.**

**Skills and confidence**

- The skills I learnt in Reboot Kids have been useful in increasing my child’s fruit and or vegetable intake.
- The home practice activities have helped me to put these skills into practice.
- I feel more confident in managing my child’s eating habits.
- The Reboot program helped to me recognize the importance of healthy eating habits for children after cancer treatment.

**Telephone sessions**

- I found the number of telephone sessions was about right.
- I would have preferred more than four telephone sessions.
- I would have preferred less than four sessions.
- I found the telephone sessions to be inconvenient.
- I found the telephone sessions to be easy to understand.
- I found the telephone sessions to be personally relevant.
- I found the telephone sessions to be useful.
- I found the telephone sessions to be too long.

**Information**

- I was satisfied with the amount of information in Reboot.
- I was satisfied with the quality of information in Reboot.
- I was satisfied with the amount of information in Reboot.

**Parent workbook**

- I found the parent workbook easy to read.
- I found the parent workbook useful.
- I will continue to use the parent workbook after the program has finished.
- The resources provided in the parent workbook were useful.

**Telephone vs online**

- I was satisfied with receiving Reboot over the telephone.
- I would have preferred to complete Reboot online.
- I would have preferred to complete Reboot online with some telephone support.
- I would have preferred to complete Reboot by myself in my own time.

**Questionnaires**

- The Reboot questionnaires were too long.
- The Reboot questionnaires were too frequent.

**Overall**

- I enjoyed participating in Reboot.
- I would recommend Reboot to other families.

## Results

Initial planning for Reboot Kids began in 2015 via a collaboration with the developers of the ‘Good for kids: Healthy Habits program’ at the University of Newcastle [15]. Healthy Habits is an evidence-based fruit and vegetable program delivered to nearly 400 Australian families. With their consent, our team updated the original parent guidebook to include the most recent version of the Australian Dietary Guidelines and Nutrition Australia Healthy Eating Pyramid, and dietitian approved recipes. To promote immediate and long-lasting intervention changes, we also followed recommendations by the original developers to focus on parent providing of fruit and vegetables to CCS, which mediated the short-and-long-term effectiveness of the intervention [22]. We therefore modified the initial behavior change technique from parenting self-monitoring of CCS daily fruit and vegetable intake to occasions of parent providing of fruit and vegetables to CCS over three days (Table 1).

Ethics approval was obtained on 11th November 2015 (HREC/15/SCHN/395) and recruitment commenced in August 2016. Of the 20 participants we aim to recruit, 7 have completed the intervention (including the booster session), 1 declined to participate after consenting, 2 participants dropped out after completing the first session, 8 are actively completing the intervention and 3 participants are providing consent. We aim to recruit the remaining participants via telephone follow-up calls and complete the intervention by July 2018.

## Discussion

This paper outlines the protocol for a pre-post, parent-led behavioral nutrition intervention for CCS, previously evaluated in a non-cancer pediatric population [15,16]. Although interventions piloted in non-cancer pediatric populations have led to significant increases in child fruit and vegetable intake [16], there is no research on their effectiveness in this high-risk population of CCS [34]; highlighting the unique contribution of the Reboot program [35].

We anticipate that the multimodal approach used in Reboot, encompassing a written parent guidebook and semi-structured telephone calls will contribute to the aim of the study. Telephone contact as the primary mode of intervention delivery can be efficacious [36], providing parents with support from a healthcare professional without requiring travel [12]. This flexibility is important in ensuring equitable access to families of CCS living in rural and regional areas [12]. Parents of CCS also report a preference for workbooks which provide relevant information about their children’s health after treatment [12].

The number of intervention contacts is also known to moderate intervention effectiveness, with behavior change often requiring multiple points of contact [37]. Booster sessions are often recommended to reinforce or re-establish messages or behavior changes suggested during interventions [38]. Subsequently, we chose to modify the original intervention to include 1 booster session. However, there is no research on the impact of booster sessions in dietary interventions in CCS. Moreover, the effectiveness [38] and optimum timing [39] of booster sessions in the wider behavior change literature is also unclear [40]. Most studies suggest that booster sessions should be instituted within three months after the intervention is complete to maximize efficacy [38,39], supporting our design of a booster session at 6 weeks post-intervention.

The Reboot study design has both strengths and limitations. As a pilot study, the small sample size will limit the ability to draw definitive conclusions regarding the efficacy of the intervention in increasing fruit and vegetable intake among CCS. The study will, however, be most useful in assessing the feasibility and acceptability of delivering a behavioral nutrition intervention in this population (Textbox 1). The inherent vulnerabilities of using parent-report as a proxy for child intake also warrants consideration [41]. A recent study indicated that repeated 24-hour recalls were a more valid measurement of dietary intake in CCS compared with FFQs, which underestimated energy intake [28]. However, due to the participant burden of multiple 24-hour recalls [42], we aimed to reduce potential bias in intake measurement by using a single 3-pass 24-hour recall and a validated, parent-administered online FFQ.

The increasing use, and success, of technology-based, parent-led interventions in improving children’s fruit and vegetable intake [43] suggests that online or smart-phone delivered interventions may offer a cost-effective alternative to telephone-based behavior change support [44-46]. Alternatively, online or mobile phone interventions delivered together with minimal telephone support or text messaging, may also help to maintain important human interaction [47] whilst still reducing intervention delivery costs. Given the absence of evidence-based dietary interventions in CCS [48], experimentation with different modes of delivery is an important next step in identifying the most efficacious method for promoting healthy eating habits in this vulnerable population [47].

Subsequently, a future goal of this pilot study is to utilize our feasibility and acceptability data to inform the development of a randomized control trial to evaluate the efficacy of delivering reboot online via web-based modules with brief telephone support (15 minutes) to reinforce key messages, on CCS dietary intake, compared with a wait-list control. If successful, data obtained from the RCT will be used to support the implementation of Reboot by community organizations across Australia, especially those in rural and remote areas, where CCS have poorer access to preventive health care [49].

## Abbreviations

CCS: childhood cancer survivors
FFQ: food frequency questionnaire
RCT: randomized controlled trial
SCH: Sydney Children’s Hospital, Randwick
